# Micro-CT – a digital 3D microstructural voyage into scaffolds: a systematic review of the reported methods and results

**DOI:** 10.1186/s40824-018-0136-8

**Published:** 2018-09-26

**Authors:** Ibrahim Fatih Cengiz, Joaquim Miguel Oliveira, Rui L. Reis

**Affiliations:** 1 3B’s Research Group, I3Bs – Research Institute on Biomaterials, Biodegradables and Biomimetics, University of Minho, Headquarters of the European Institute of Excellence on Tissue Engineering and Regenerative Medicine, AvePark, Parque de Ciência e Tecnologia, Zona Industrial da Gandra, Barco, 4805-017 Guimarães, Portugal; 2 ICVS/3B’s – PT Government Associate Laboratory, Braga/Guimarães, Portugal; 3 The Discoveries Centre for Regenerative and Precision Medicine, Headquarters at University of Minho, AvePark, Parque de Ciência e Tecnologia, Zona Industrial da Gandra, Barco, 4805-017 Guimarães, Portugal

**Keywords:** Systematic review, Microstructure, Mineral density, Scaffolds, Tissue engineering

## Abstract

**Background:**

Cell behavior is the key to tissue regeneration. Given the fact that most of the cells used in tissue engineering are anchorage-dependent, their behavior including adhesion, growth, migration, matrix synthesis, and differentiation is related to the design of the scaffolds. Thus, characterization of the scaffolds is highly required. Micro-computed tomography (micro-CT) provides a powerful platform to analyze, visualize, and explore any portion of interest in the scaffold in a 3D fashion without cutting or destroying it with the benefit of almost no sample preparation need.

**Main body:**

This review highlights the relationship between the scaffold microstructure and cell behavior, and provides the basics of the micro-CT method. In this work, we also analyzed the original papers that were published in 2016 through a systematic search to address the need for specific improvements in the methods section of the papers including the amount of provided information from the obtained results.

**Conclusion:**

Micro-CT offers a unique microstructural analysis of biomaterials, notwithstanding the associated challenges and limitations. Future studies that will include micro-CT characterization of scaffolds should report the important details of the method, and the derived quantitative and qualitative information can be maximized.

## Background

In nature, structural materials including animal and human tissues have complex hierarchical architectures at multiple scales from nano to macro [1]. To achieve the regeneration of functional tissues, as in nature, complete understanding and biomimicry of those 3D architectures are necessary. Differences in the design of 3D scaffolds [2] such as composition, surface chemistry, architecture, and mechanical properties, can yield to almost countless different scaffolds. Scaffolds host and interact with cells, and the design of a scaffold affects the entire behavior of the cells including adhesion, growth, migration, differentiation, and matrix synthesis [3–8]. Certainly, the functional performance of a scaffold *in vivo* not only depends on its microstructure but also on all other factors involved in tissue engineering which are very complex and probably not yet completely known [9].

Tomography is defined as a method by which an object’s 3D image that corresponds to its internal structure is obtained. Micro-computed tomography (micro-CT) [10, 11] is a high-resolution CT that has a pixel size typically between 1 μm and 50 μm, and allows to investigate the microstructure of samples using X-rays. Conventionally, the samples can be analyzed almost without any sample preparation process generally in a non-destructive way. Today, a search on “micro-CT” in PubMed yields more than 10,000 items by being used in many fields. Within the field of tissue engineering it has a place in many application domains including (i) scaffold characterization [12–15], (ii) *in vivo* small laboratory animal tissue characterization [16–19] including assessment of bone turnover using 4D micro-CT data [20], and tumor detection [19], (iii) *ex vivo* characterization of human tissues [21, 22] and animal tissues [23–27]. Micro-CT has frequently been used in bone studies, and typically, the investigated parameters include volume, microstructural features, and mineral density. The investigation of soft tissues is relatively challenging due to their low contrast in conventional micro-CT imaging; thus it may require an extra effort such as employing high-atomic-number element probes [28] or contrast agents [29–32].

### 3D Microstructural metrology of scaffolds used in tissue engineering with tomography

**Fig. 1:**
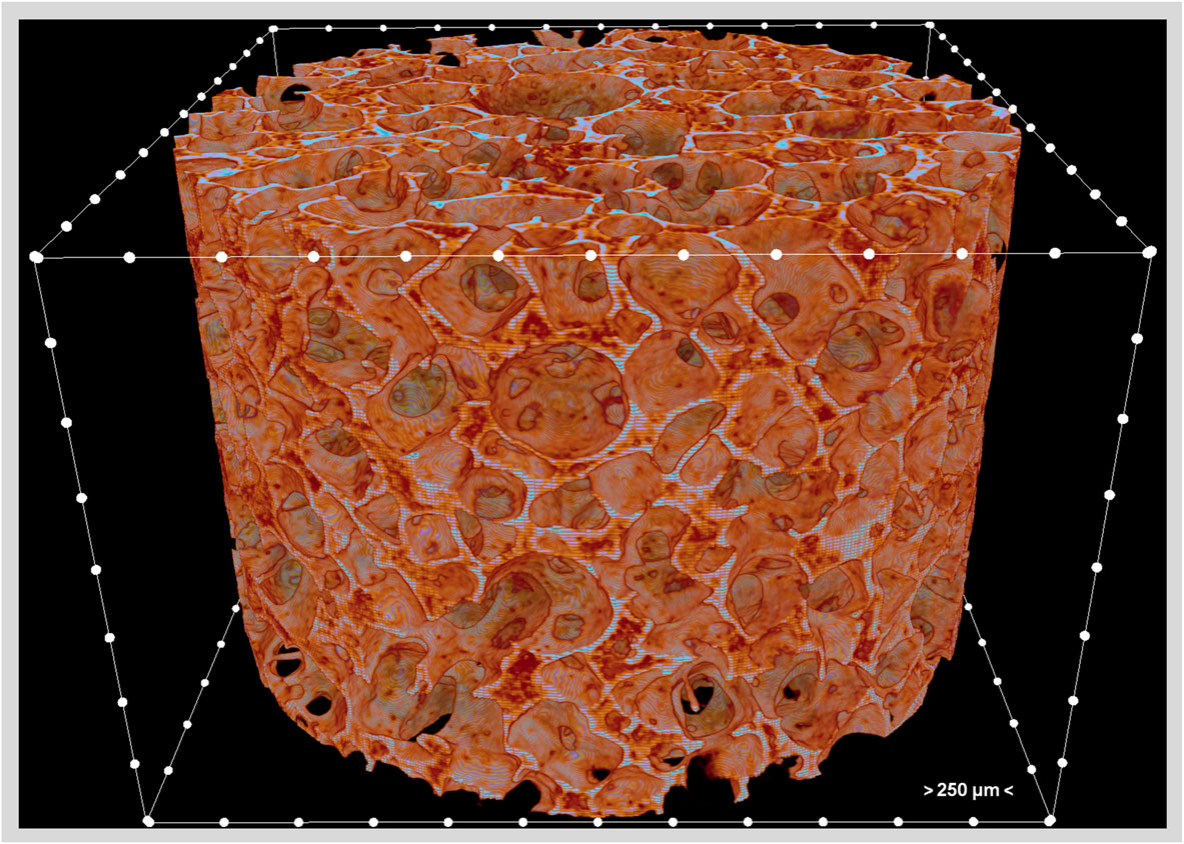
A 3D micro-CT image of the polycaprolactone-polyurethane scaffold. The distance between two adjacent white dots is 250 μm

Given the critical role of microstructure on the performance of scaffolds, characterization of the microstructure is indispensable, and Micro-CT is an outstanding instrument to characterize the microstructure of scaffolds. A large surface area assists cell attachment and proliferation. Porosity and pore size define the surface area per volume (Fig. 1) [33]. High porosity assists nutrient and waste diffusion that is one of the critical factors for vascularization and tissue ingrowth [34, 35]. Additionally, porosity needs to be controlled for each application since an increase in porosity leads to a relative decrease in the mechanical properties [36]. The interconnection of the pores is also an important parameter that defines the effects of porosity and pore size [35, 37]. Given a scaffold with constant porosity, a decrease in the mean pore size will cause pore channels to narrow, which may inconvenience cell migration despite an increase in the surface area [38]. Relatively smaller pores and larger specific surface area assist cell attachment as shown in a study with collagen-glycosaminoglycan scaffolds that attachment of osteogenic cells was increased with decreasing mean pore size and increasing specific surface area [39]. Regarding the mobility of cells in a scaffold, it is not affected by the pore size alone, but also scaffolds’ mechanical properties and adhesiveness [40]. Cell arrangement and morphology are influenced by scaffolds’ architecture and topography [41], and it was shown that cell morphology has a role in the cell function [42–45]. Surface roughness may lead cells to have a round morphology while flat surfaces lead to an increased cell spreading [41]. Presence of aligned structures can facilitate cell alignment, for example, designing concave microgrooves in scaffolds can also be effective to align cells, and has implications in muscle tissue engineering by facilitating the formation of layered bundle tissues [46].

In a study with osteoblast-like cells, increased cell ingrowth was observed in the scaffolds with relatively smaller pores among the scaffolds with different mean pore sizes ranging from 100 μm to 200 μm [47]. In another study, a scaffold with gradually changing pore sizes from 88 μm to 405 μm was examined, and the pore range of 380–405 μm provided higher chondrocyte and osteoblast proliferation while the range between 186–200 μm was more suitable for fibroblast proliferation [48]. On the other hand, new bone formation occurred faster in the scaffolds with the pore size range of 290–310 μm than in the other scaffolds [48]. Scaffolds with a mean pore size of 250 μm had higher fibrous tissue ingrowth *in vivo* than that of other scaffolds with mean pore sizes of 30 μm, 60 μm, 110 μm, 350 μm and 700 μm [38]. The scaffolds with mean pore sizes of 30 μm and 60 μm had no tissue ingrowth even though pore sizes are larger than the size of a fibroblast [38]. The size of the pores can affect the osteogenic differentiation and proliferation of stem cells oppositely [49]. Ceramic scaffolds with a pore size of 200 μm and 500 μm were found to provide a relatively higher rate of osteogenic differentiation, and proliferation, respectively, when compared to each other [49]. Both surface chemistry and pore size can significantly affect the lineage-specific differentiation of stem cells while the effect of surface chemistry found to be relatively larger if the pore size is smaller than 300 μm [50].

**Fig. 2:**
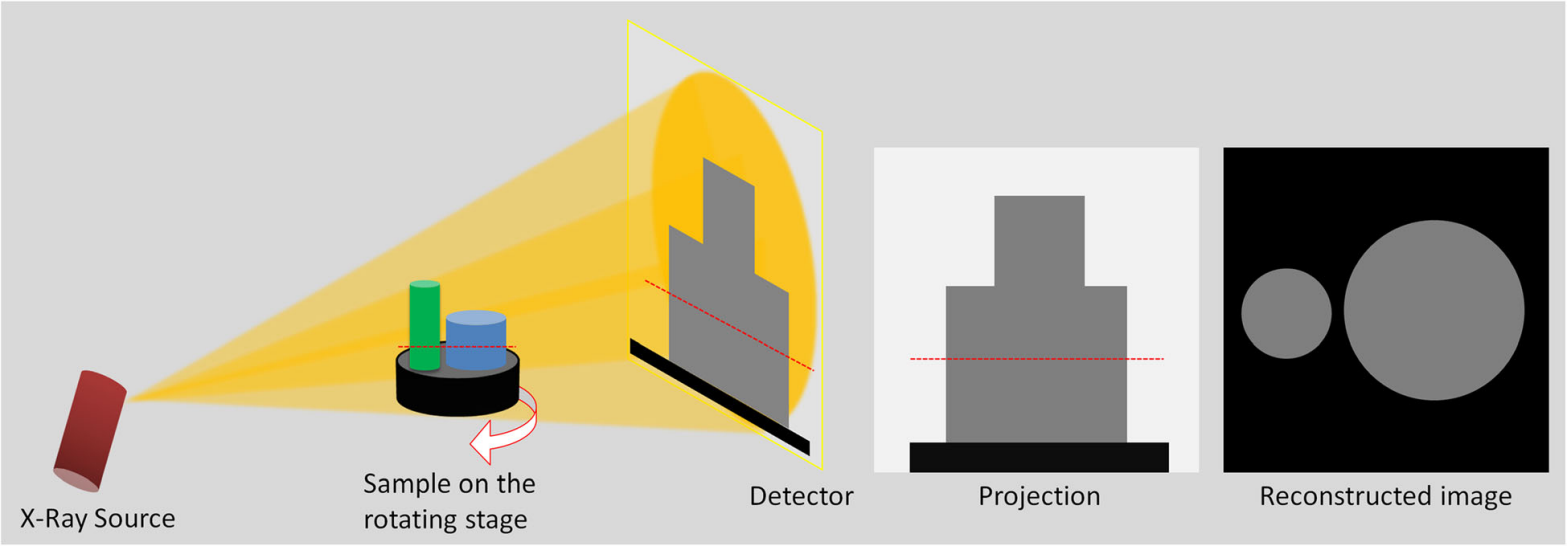
Schematic illustration showing the basics of micro-CT. Cone-beam X-rays travel from the source to the detector through the sample with attenuation, and a gray-scale projection image is acquired at each rotation angle. Projection images are then reconstructed, and the reconstructed image dataset is used for analysis. The red dashed line indicates the vertical position of the reconstructed image, *i.e.*, the cross-sectional image

**Fig. 3:**
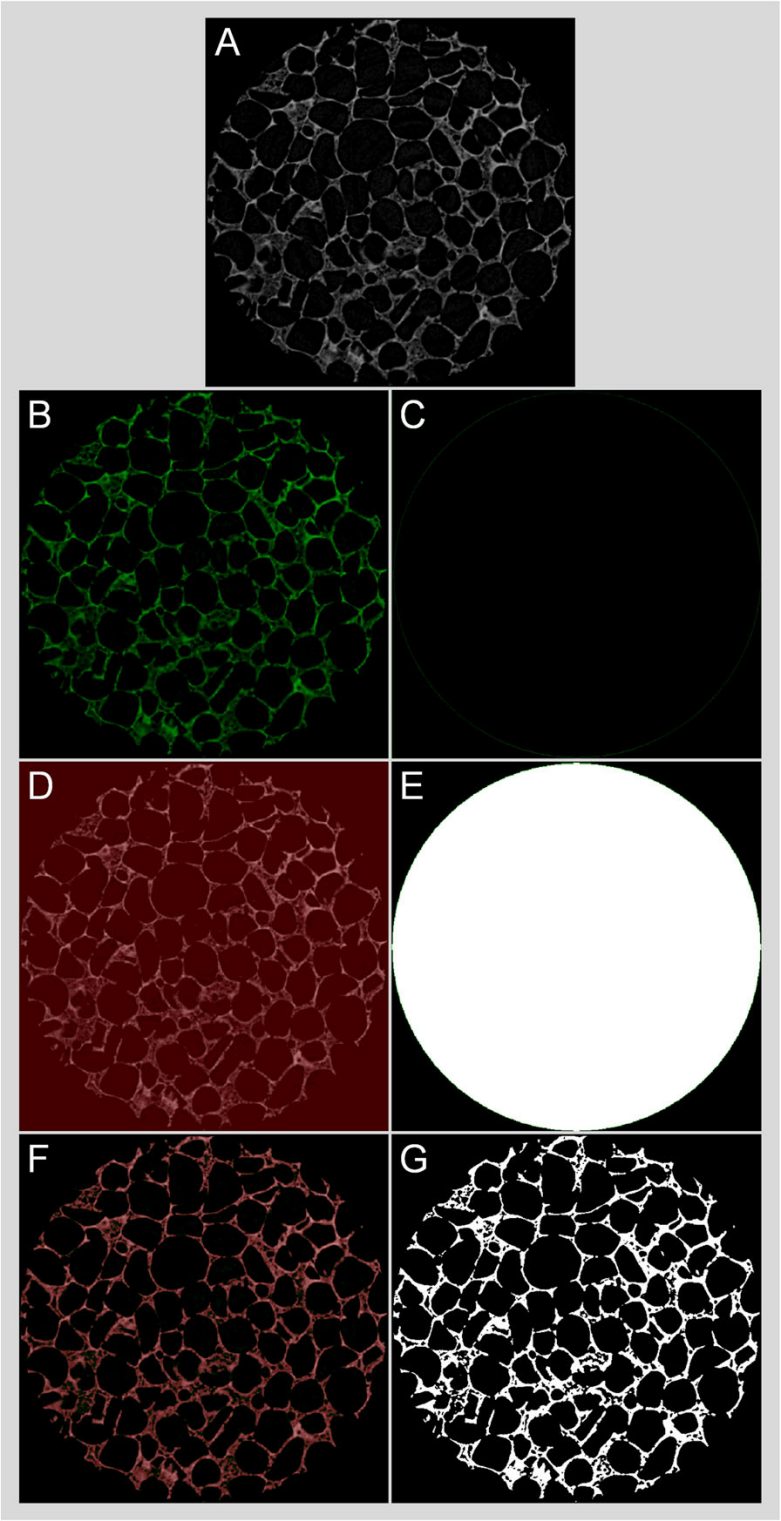
Binarization of reconstructed micro-CT images. Gray-scale reconstructed 2D image of a silk-based tissue engineering scaffold with a circular region of interest (ø 3 mm) (**a**), half-tone views (**b**, **d**, and **f**) and the corresponding binary images (**c**, **e**, and **g**) respectively if no gray-scale value is included that yields to complete black, if entire gray-scale values are included that yields to complete white, and if the right gray-scale values (that is in this case 38-255) are obtained by global thresholding that yields to a binary image showing the microstructure of the scaffold

Micro-CT characterization comprises three major sequential processes: acquisition, reconstruction, and analysis [13]. The X-rays are generated by the source and emitted to the target sample. When passing through the sample, the X-rays are attenuated based on the properties of a sample that is being scanned (*e.g.*, its density, thickness, and composition). The acquisition is completed by collecting 2D projection images (radiographs) from many viewing angles. In the conventional micro-CT instruments, the X-ray system is fixed, and the sample stage rotates, while in the instruments for live small animals the stage is fixed, and the X-ray system rotates to scan the animal. The projection images are reconstructed by a computer using algorithms [51–53] to obtain the cross-sectional 2D images in the transverse plane (Fig. 2). Binarization is the process by which the pixels that belong to sample are discriminated in the reconstructed images that are in a contrast scale of 0-255, and the images are made black and white (Fig. 3). Typically, white indicates the material while black indicates empty space. Volume, porosity, pore size, and suchlike results can be quantified using the binary image dataset. Commercial micro-CT instruments come with the manufacturers’ software while an external software such as ImageJ (https://imagej.nih.gov/ij/index.html) could also be used.

### Systematic search of the papers that were published in 2016

**Fig. 4:**
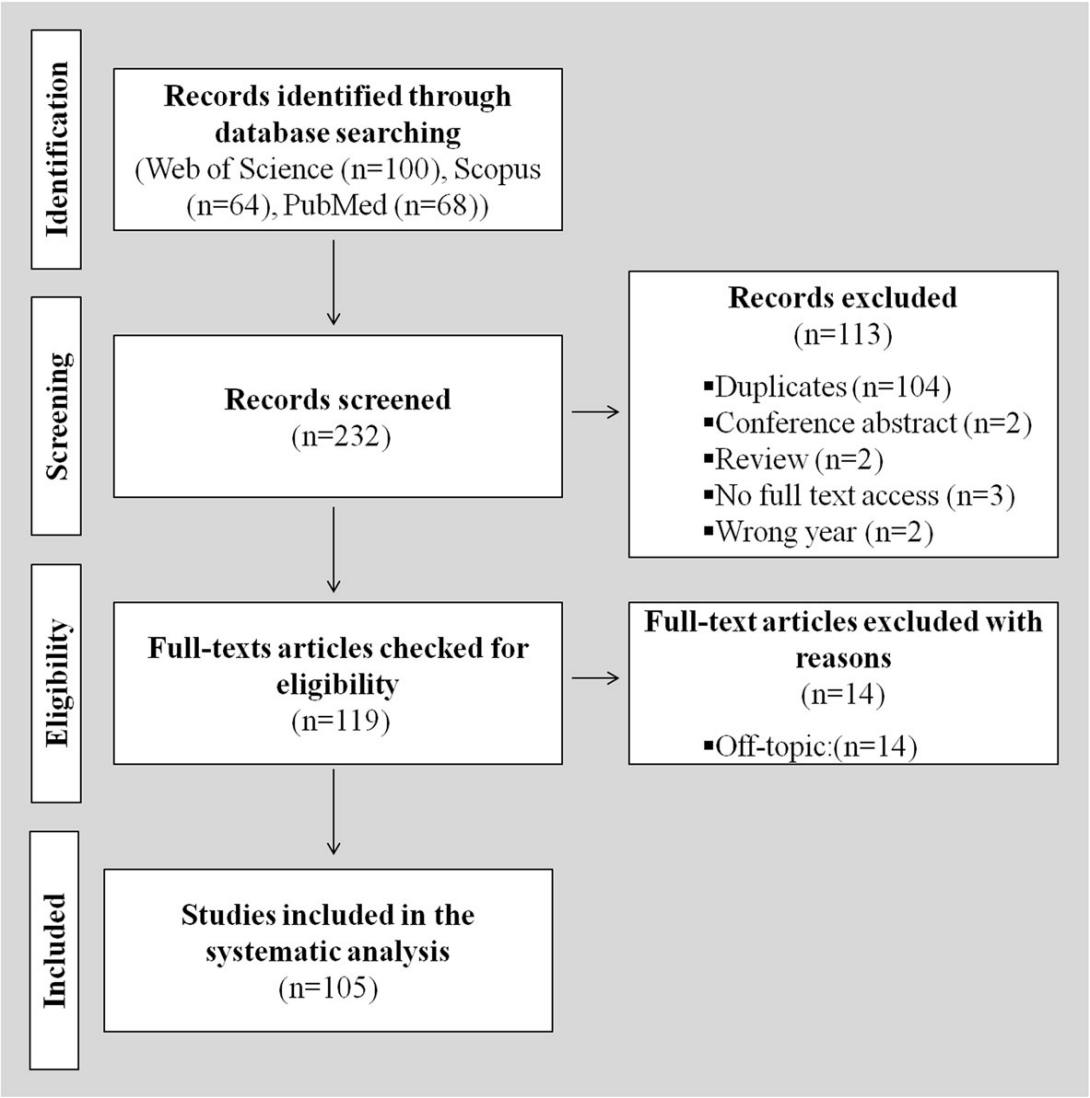
The flowchart of the systematic search

A systematic search was performed on the papers that were published in a predetermined year, 2016, to answer these questions: (i) what type of data was reported in the papers, *i.e.*, quantitative or qualitative, or both, (ii) what kind of quantitative results were reported (such as microstructure (*e.g.*, porosity, pore size, wall thickness), volume, and mineral density if relevant), and (iii) whether the reporting of the methods were adequate including the information on pixel size, number of replicates, rotation step or number of obtained projections, voltage and current values, and use of any filter). A search was performed on the electronic databases of Scopus, Web of Science, and PubMed using the term “micro-CT” with “scaffold” to identify the relevant original research papers in English that were published in 2016. The year 2016 was selected in the current work because it was presumed as a representative period. The papers that involve micro-CT characterization of scaffolds were considered as eligible. The papers were screened, selected as shown in the flowchart that is presented in Fig. 4, and analyzed to answer the aforementioned questions.

### Findings of the systematic search and discussion

**Fig. 5:**
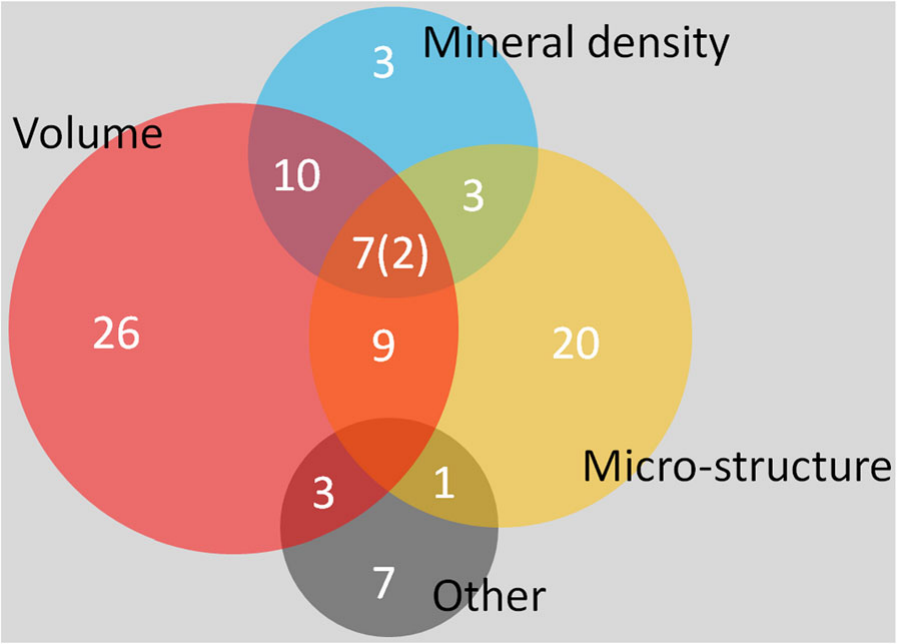
Venn diagram showing the number of the papers that reported quantitative results regarding the volume, microstructure, mineral density and other measures. Two of the seven papers that reported volume, microstructure, and mineral density also reported additional results. The sizes of the circles in the diagrams are directly proportional to the number of the associated papers

A total of 105 papers [54–158] were included in the systematic analysis. The data indicated that micro-CT was used only qualitatively in around 15% of the papers [63, 74, 75, 79, 95, 102, 106, 108, 112, 116, 122, 129, 133, 146, 149, 158], and only quantitatively in 9.5% of the papers [55, 78, 83, 85, 98, 111, 125, 131, 137, 155]. Micro-CT was used both qualitatively and quantitatively in the rest of the papers [54, 56–62, 64–73, 76, 77, 80–82, 84, 86–94, 96, 97, 99–101, 103–105, 107, 109, 110, 113–115, 117–121, 123, 124, 126–128, 130, 132, 134–136, 138–145, 147, 148, 150–154, 156, 157]. Only around 29% of the papers reported the number of replicates (*i.e.*, “n”) they analyzed, and around 77% of the papers reported the used pixel size. Among the papers that reported n, the mean size of n was 4.4 with a standard deviation (SD) of 2.8. The mean used pixel size was 16.8 μm with an SD of 13.3. The analysis also showed that less than 3% of the papers used the term “spatial resolution”, and around 14% of the papers used the term “resolution” which might get confused with “pixel size” as it was discussed below. As Fig. 5 illustrates, volume measurements, microstructural characterization, and mineral density determination are the three most frequent quantitative results. The results categorized as “other” include histograms, area/distance measurements such as tissue thickness, bone contact area or graft diameter, callus index and intensity signals. The rotation step, or the number of obtained projections, is not mentioned in over 68% of the papers. The information on the used voltage and current was not reported in around 26% of the papers, while around 73% of the papers did not report whether they used a filter or not. One paper was identified that the term “*in vivo* imaging” was used when only explants were characterized.

The internal architecture of a scaffold has major roles in the cell behavior and thus on the overall performance of the construct, the characterization of its macro (> 100 μm) and micro (0.1 μm - 100 μm) structure is inherently of interest. With the demonstrated use and advantages of micro-CT, it has been a valuable instrument in research laboratories with its challenges that are summarized in Table 1.

**Table 1:** Challenges in the conventional micro-CT characterization

Challenge	Potential solution	Reference
Artifacts and noise.	Detailed in Table 1 of Ref. [162]	[162, 164, 169, 170]
Soft tissue characterization is not as *easy* as a dry-state porous scaffold.	Using contrast agents or high-atomic-number element probes can help.	[28–32]
Operator-determined acquisition parameters may affect the results.	Parameters should be optimized during the preliminary study.	[13]
Harsh acquisitions may damage/alter the sample/tissue (for example, discoloring of a biomaterial or tumorigenesis in animals); and radiation exposure of animals in live animal studies, lethal dose 50/30 (both ethical and scientific considerations).	Long scans (either due to very low rotation step, frame averaging, or long exposure time) should be avoided, and/or X-ray energy could be decreased.	[11, 159–161]
Comparing micro-CT results of different studies is not easy if the used parameters are not identical.	There is a need to establish a protocol with determined values for parameters.	[13]
Issues with very dense/thick samples resulting in a dataset of almost only black images.	This is because there will be no contrast since no X-ray can pass; however, use of a filter may resolve the problem, but it may greatly increase the acquisition time. The sample can be cut to its smaller representative volume. If it is not possible, then another instrument could be used such as a scanning electron microscope.	
Issues with very thin/light samples or a hydrogel, then no contrast will be obtained (this gives a dataset of images with very low contrast).	Contrast agents can be used.	[29–32]
A limited volume of sample that can be analyzed at once (the images have a certain number of pixels with a certain size of a pixel).	Display matrix size and/or pixel size can be adapted. The representative portion of the sample can be pre-determined.	
Overlaps in gray-scale values in multi-material samples (*i.e.,* scaffold-bone explants or composites).	Advanced segmentation protocol can be used.	[171]
Considerations on the maintenance and sharing of micro-CT data.	During the preliminary study, the duration of the micro-CT characterization and the disc space requirements can be estimated.	[13]

Micro-CT has been considered as the gold standard for bone explants’ microstructure and morphology study [25]. In the case of scanning live animals, excessive exposure to radiation [159–161] can be a problem for the survival of the animal, tumorigenesis, and ethics. Using a pixel size less than 50 μm may be fine to prevent excessive exposure to radiation [11].

From the results of this systematic analysis, two major points can be emphasized to be resolved in future studies that will use micro-CT: (i) methods section from acquisition to analysis should be complete, and (ii) information obtained should be maximized. The methods part should cover the used pixel size, voltage and current, number of replicates, scanning medium, use of any filter, rotation step/number of projections, and use of any specific image processing. Various results can be derived from a Micro-CT characterization. These results can be quantitative or qualitative, and obtained in a 2D or 3D fashion. Qualitative results include X-ray and reconstructed image of the sample, mineral density color map, pores size color map, and structure thickness color map, and thus, provide valuable information. Quantitative results include quantification/proportion of volumes, microstructural features (mean pore size, mean wall thickness, and their distribution and change vertically across the sample), mineral density, histograms, quantification of surface area, and surface:volume ratio. It should be noted that some of these parameters are relevant only for certain studies. For instance, bone mineral density is related only with scaffolds for bone tissue engineering. Therefore, this is a factor affecting the outcome of the systematic analysis.

CT experts may accept that resolution is a quite sensitive topic among authors by being a measure of image quality. As the outcome of the systematic analysis shows, some authors including some of the CT experts prefer to use terms “resolution” and “spatial resolution” instead of “pixel size” or “voxel size”, while some micro-CT system manufacturers prefer to communicate the size of the pixels or voxel of the images. Additionally, there is no standardized approach for spatial resolution reporting accepted by the micro-CT system manufacturers [25]. The definitions of these terms are presented in Table 2.

**Table 2:** Terms that are associated with the micro-CT images, and their definitions

Term	Definition
Pixel size	Size of the 2D discrete parts that make up a 2D micro-CT image. Usually, expressed as a single value, *e.g*., 10 μm, indicating the size of each pixel is 10 μm × 10 μm.
Voxel size	The 3D equivalent of pixel size indicates the size of each voxel, Typically, micro-CT images isotropic (identical size in all dimensions), *e.g*., 10 μm × 10 μm × 10 μm, in which case pixel size and voxel size provide identical information.
Resolution	▪ Smallest perceptible detail (complete and exact definition can be found in Ref. [164]) ▪ Resolution of an image can indicate the number of pixels in an image (such as 800 × 600). The second definition is referred to the display matrix size in the standard guide of ASTM International: E1441-11.
Spatial resolution	▪ Smallest displacement that can be measured in the measurement direction [164] ▪ Smallest separation where two points can be identified as separate parts (complete and exact definition can be found in the standard guide of ASTM International: E1441-11)

The studies that report a value for resolution need to show how they quantify the resolution whether it is the pixel size given by the scanner software or they quantified it with calibrated thickness wires [162]. Given the definitions of the terms, the images that have the same number of pixels would contain different information if their pixel sizes are not identical. It is noteworthy to know that a smaller pixel size does not necessarily provide a better image, for instance, the image does not improve when the pixel size is smaller than the X-ray spot size [162, 163]. Some other factors [25, 162, 164, 165] are needed to be considered as well, regarding X-ray (*e.g.*, energy, geometry, and focal-spot size), sensitivity of detector, use of filter, integration time of X-ray with the sample per projection, characteristics of the sample (*e.g.*, composition and size), scanning medium, noise and artifacts. The attenuation of the X-rays depends on the characteristics of the sample such as its density and thickness. The level of the X-ray energy, *i.e.*, the source voltage and current, should be tuned for the sample (denser samples require higher energy) since the obtained the images are in gray-scale and associated with the X-ray absorption that is linked with the sample’s electron density [163]. Therefore, the reconstructed images are quantitatively densitometric. The pixel size and rotation step can have significant influences on the quantitative and qualitative results of the μ-CT characterization of scaffolds, as well as the size of the generated data and the duration of the characterization [13]. Integration time is the duration of each projection. A longer integration time provides more photos to be detected and affect the micro-CT image [166]. The use of a filter can improve the characterization of thick/dense samples by attenuating the low-energy X-rays and minimizing the beam hardening effect [25]. Spatial resolution depends on the geometry of the X-ray, and detector [10]. Cone beam X-rays and flat panel detectors provide significantly shorter scanning times because the data for multiple slices is obtained in each rotation step, while fan beam X-rays and linear detectors provide less scatter effects for thick samples [167] and higher accuracy [164]. Reconstruction method also depends on the geometry of the source. The scanning medium (*e.g.*, air, water, phosphate-buffered saline, or ethanol) influences the characterization results since different media affect the X-ray attenuation differently [25, 168].

## Conclusion

The microstructure of tissue engineering scaffolds greatly influence the behavior of cells, and the performance of the tissue engineering construct; therefore, the characterization of the scaffolds’ microstructure is of keen interest. Micro-CT is an outstanding instrument for the quantitative 2D and 3D analysis and visualization of scaffolds. The analysis results showed that the methods sections of the majority of the analyzed papers are in need of improvement in reporting the details of micro-CT characterization. Moreover, the amount of quantitative and qualitative information from micro-CT characterization can be maximized. Given the fact that the obtained results from micro-CT characterization significantly depend on several parameters, the important acquisition related details should be clearly provided in the papers. It is recommended that the parameters include the used pixel size, rotation step, X-ray energy, scanning medium, use of filter, as well as the number of samples analyzed since the omitted data complicate the reproducibility of the experiments.
